# PBC-Transformer: Interpreting Poultry Behavior Classification Using Image Caption Generation Techniques

**DOI:** 10.3390/ani15111546

**Published:** 2025-05-25

**Authors:** Jun Li, Bing Yang, Jiaxin Liu, Felix Kwame Amevor, Yating Guo, Yuheng Zhou, Qinwen Deng, Xiaoling Zhao

**Affiliations:** 1 College of Information Engineering, Sichuan Agricultural University, 46 Xinkang Road, Yucheng District, Ya’an 625000, China; lijun@sicau.edu.cn (J.L.); 2023219006@stu.sicau.edu.cn (B.Y.); jiaxin10988@gmail.com (J.L.); 2024319029@stu.sicau.edu.cn (Y.G.); 2024319036@stu.sicau.edu.cn (Y.Z.); 2023319027@stu.sicau.edu.cn (Q.D.); 2 Agricultural Information Engineering Higher Institution Key Laboratory of Sichuan Province, Ya’an 625000, China; 3 Ya’an Digital Agricultural Engineering Technology Research Center, Ya’an 625000, China; 4 Key Laboratory of Livestock and Poultry Multi-omics, College of Animal Science and Technology, Sichuan Agricultural University, Chengdu 611130, China; amevorfelix@gmail.com

**Keywords:** poultry behavior classification, image captioning, transformer, concentrated attention, positional encoding

## Abstract

Accurate classification of poultry behavior is critical for assessing welfare and health, yet most existing methods predict behavior categories without providing explanations for the image content. This study introduces the PBC-Transformer model, a novel model that integrates image captioning techniques to enhance poultry behavior classification, mimicking expert assessment processes. The model employs a multi-head concentrated attention mechanism, Head Spatial Position Coding (HSPC), to enhance spatial information; a learnable sparse mechanism (LSM) and RNorm function to reduce noise and strengthen feature correlation; and a depth-wise separable convolutional network for improved local feature extraction. Furthermore, a multi-level attention differentiator dynamically selects image regions for precise behavior descriptions. To balance caption generation with classification, we introduce the ICL-Loss function, which adaptively adjusts loss weights. Extensive experiments on the PBC-CapLabels dataset demonstrate that PBC-Transformer outperforms 13 commonly used classification models, improving accuracy by 15% and achieving the highest scores across image captioning metrics: Bleu4 (0.498), RougeL (0.794), Meteor (0.393), and Spice (0.613).

## Simple Summary

Farmers and animal welfare experts need clear and reliable tools to understand what chickens and other poultry are doing so they can spot health or welfare problems early. In this study, we created a new AI model that not only tells you which behavior it sees—such as eating, resting, or moving—but also explains in everyday language what in the image led to that decision. We tested this model on thousands of real poultry images and found that it is much more accurate than twelve commonly used methods and gives very clear, human-like descriptions of each behavior. By combining both identification and explanation, this approach can help those who care for poultry monitor their flocks more effectively, improve everyday management, and ultimately support better health and welfare for the animals.

## 1. Introduction

Behavior analysis is essential for evaluating animal health and welfare, aiding farm personnel in monitoring livestock conditions [1]. Traditional behavior classification methods fall into invasive and non-invasive categories [2]. While invasive methods improve identification accuracy using external markers, their high cost and potential stress on animals limit their practical application [3]. Non-invasive approaches, such as video-based tracking, minimize interference but often require labor-intensive manual analysis [4].

Advancements in deep learning have revolutionized poultry behavior analysis. Early studies employed image processing algorithms, later transitioning to deep learning techniques [5,6,7]. For instance, Lin et al. [8] used CNNs to monitor broilers’ heat stress responses, while Okinda et al. [9] developed a 2D posture descriptor-based system for disease detection. Other studies have integrated object detection (YOLOv3) and classification models (ResNet50) to enhance accuracy. However, most methods rely on single-modal visual features, neglecting contextual information and interpretability [10]. Furthermore, Cuan et al. [11] proposed a CNN-based approach to analyze acoustic features of sick and healthy chickens to achieve promising detection rates.

Despite these advancements, most current research on poultry behavior analysis remains focused on extracting single-modal features, which presents certain limitations in decision-making. Visual features often emphasize pixel-level or object-level data but neglect contextual information, and extracting acoustic features can be challenging due to the need to suppress background noise [12].

Furthermore, these single-modal approaches struggle to establish a strong link between classification decisions and their underlying rationale, limiting their practical value. Furthermore, high-performing classification models often require vast amounts of training data, and in cases where poultry behaviors are similar, the accuracy of CNN/Transformer-based models decreases. On the other hand, agricultural experts first observe behavior, focus on key features, recall past cases, and then make decisions, an approach that allows them to easily distinguish between similar behaviors, something CNN/Transformer models struggle with.

In summary, poultry behavior classification currently faces three key challenges:

1. How can we replicate the expert classification process comprising observation, focus, recall, description, and decision-making when poultry behavior categories are similar, rather than simply offering a decision?
2. How can we improve classification accuracy when data are limited or behavior categories are imbalanced?
3. How can we overcome the challenge of static vectors, which prevent models from dynamically selecting image regions to generate accurate descriptions, especially in images with many irrelevant elements?

To address these challenges, we propose integrating image captioning to enhance model interpretability. Image captioning generates natural language descriptions that contextualize classification decisions, closely resembling expert assessment. In addition, multi-task learning (combining classification and captioning) mitigates data scarcity issues, while a concentrated attention mechanism and multi-stage attention differentiator refine feature extraction [13]. Finally, the proposed concentrated attention mechanism and multi-stage attention differentiator can dynamically identify relevant regions within poultry images, refine attention features, and filter out irrelevant areas, addressing the third challenge.

The key contributions of this study are as follows:

- The development of the first dual-task dataset, PBC-CapLabels, for poultry behavior classification and description.
- The introduction of a novel concentrated attention mechanism (HSPC+LSM+KFE) that enhances the ability of the model to locate and extract detailed local features of poultry.
- The proposal of a novel multi-stage attention differentiator that overcomes the limitation of static vectors by dynamically selecting different regions to generate accurate descriptions.
- The development of a multi-stage attention memory report and classification decoder to ensure persistent feature memory and alignment of textual and categorical information.
- The proposal of an enhanced contrastive loss function that integrates contrastive learning of category, text, and image feature losses.

## 2. Methods

### 2.1. General

**Figure 1:**
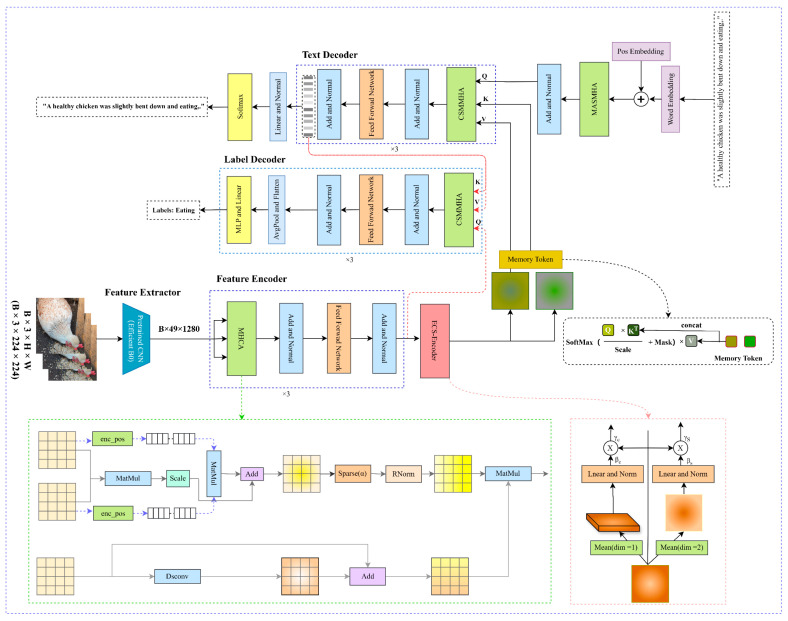
Architecture of the PBC-Transformer. Note: Contains five major modules: feature extractor, feature encoder, ESC encoder, label decoder, and text decoder.

The baseline model in this study is based on the “encoder–decoder” architecture introduced by Vinyals et al. [14]. The PBC-Transformer is an end-to-end dual-task model designed for simultaneous poultry behavior classification and text generation, as illustrated in Figure 1. It consists of five key components: an image feature extractor, a concentrated encoder, a multi-stage attention differentiator, a description decoder, and a classification decoder. Initially, poultry images are processed through the feature extractor, generating global feature vectors. These vectors serve as the Key (K), Value (V), and Query (Q) inputs for the feature encoder, which consists of three stacked Transformer layers that produce refined attention features. The multi-stage attention differentiator then enhances these features by refining spatial and channel attention, while also generating text features that serve as K, V, and Q inputs for the three-layer description decoder. The description decoder generates text sequences by initiating with a [start] token and continuing until it reaches the [end] token, predicting the next word in the sequence iteratively. During the testing phase, the model generates word vectors from the [start] token until it completes the sentence. The output of the description decoder, referred to as text-weighted visual features, is then used as K and V inputs in the three-layer classification decoder, while the global visual feature vector serves as Q. The classification decoder ultimately predicts the poultry behavior category and its corresponding textual description.

To enhance differentiation between the subject and the background, the original multi-head attention (MHA) mechanism was replaced with multi-head concentrated attention (MHCA) in the encoder. Additionally, a spatial-channel attention differentiator was introduced to dynamically identify relevant image regions, improving the accuracy of sentence generation. Inspired by Burtsev et al. [15], memory units were incorporated into the decoder to simulate the expert-like process of recalling similar cases before making a decision, thereby enhancing the model’s ability to manage global context. Figure 1 visually represents the concentrated attention mechanism, attention differentiator, and memory units.

### 2.2. Feature Extractor

For feature extraction, we used EfficientNetB0 [16], pre-trained on ImageNet [17], as the backbone network. Since this phase focuses solely on extracting image features, we omitted the final pooling and linear layers of EfficientNetB0 and fine-tuned select weights.

**Figure 2:**
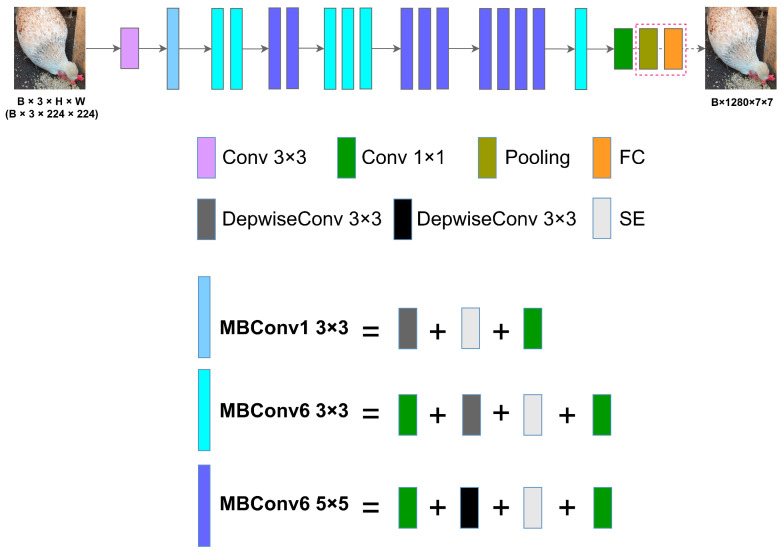
Framework of the image feature extractor. Note: Different colored modules represent different operations.

Figure 2 shows the structure of the feature extractor, where different colored blocks represent distinct operations. The notation Conv3×3 indicates a 3×3 convolution paired with a SiLU activation function and batch normalization (BN) layer. The MBConv block consists of three substructures: MBConv1×1, MBConv3×3, and MBConv5×5, where the numbers 3 and 5 represent the kernel sizes in the depth-wise separable convolutions. Modules of the same color represent identical operations.

### 2.3. Concentrated Encoder

**Figure 3:**
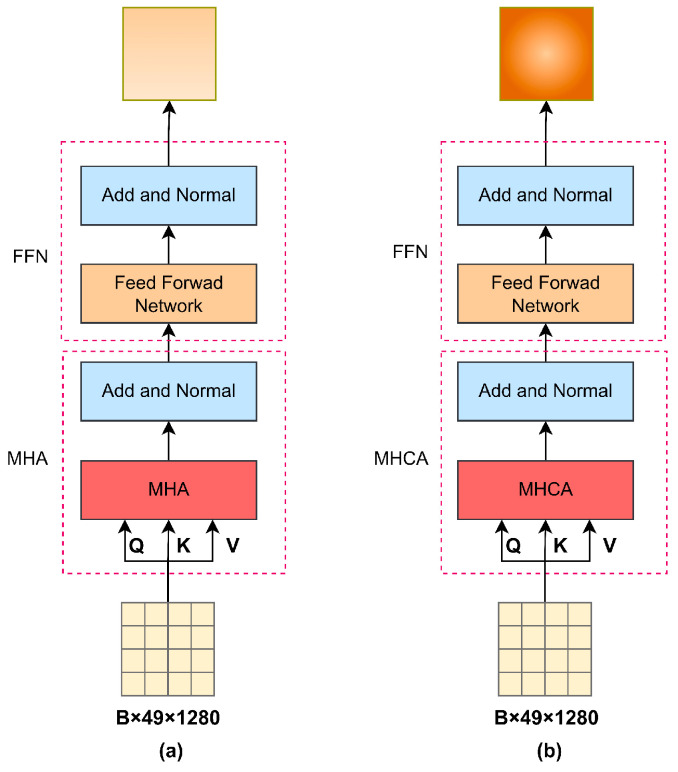
Comparison of encoders. Note: (**a**) represents the standard Transformer as an encoder and (**b**) represents our proposed concentrated encoder. The main difference between the two is the use of MHCA and MHA.

The feature encoder of the PBC-Transformer follows a structure similar to the baseline model but incorporates stacked concentrated Transformer layers. Instead of standard Transformer layers, it consists of three layers of multi-head concentrated attention (MHCA) combined with a Feed-Forward Network (FFN). The core of this feature encoder is the concentrated attention mechanism, designed to enhance the representation of poultry visual features. Figure 3 compares the structures of the standard Transformer encoder (a) and the concentrated attention encoder (b).

The concentrated attention mechanism employs a novel positional encoding method to better capture spatial and channel positional information. To distinguish between poultry and their surroundings, a sparse mechanism is introduced. In addition, to address the limitations of Transformers in representing local features, a depth-wise separable convolution is incorporated into the K-dimension of the multi-head concentrated attention.

#### 2.3.1. Concentrated Attention Mechanism

Thus, the steps of the concentrated attention mechanism are shown in Algorithm 1:
**Algorithm 1**: Concentrated attention with **HSPC**, **LSM**, and **KFE.**Input: information matrices: Q**,** K**, and** V. Output: **output__CA_**_,_**attn.**Steps:   1. Head Space Position Coding (**HSPC**):      - Compute initial attention scores.      - Apply positional encoding and update scores.      2. Limited Softmax Masking (**LSM**):      - Apply the attention mask if provided.      - Perform top-k filtering on scores.      - Apply softmax and dropout.      - Compute the context vector.      3. Kernel Fusion Enhancement (**KFE**):      - Apply depth-wise separable convolution to value matrix.      - Combine the context vector with convolved values.

#### 2.3.2. Head Space Position Coding (HSPC)

**Figure 4:**
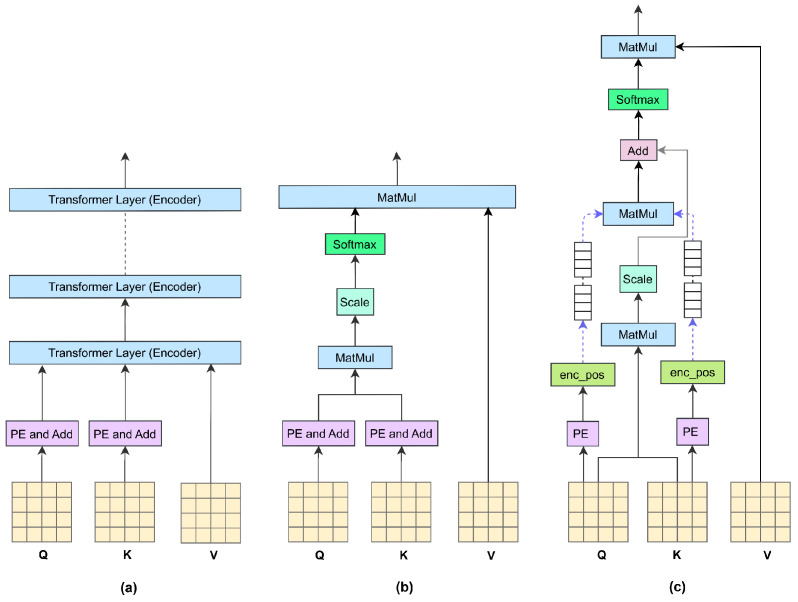
Comparison of positional encoding methods. Note: (**a**) represents the position coding method of the original Transformer, (**b**) represents the improved additive coding method, and (**c**) represents the multiplicative coding method proposed by us.

As shown in Figure 4a, the standard Transformer layer directly integrates positional encoding with input features. However, in multi-head attention mechanisms, each attention head (Q, K, and V) independently computes self-attention, limiting the ability of positional encoding to fully capture spatial relationships between different heads. This limitation becomes more pronounced when the inputs for Q, K, and V differ, as conventional positional encoding constrains the rank of the attention matrix, thereby restricting the model’s capacity to extract detailed visual information. To address this issue, recent studies have proposed various improvements. As illustrated in Figure 4b, Chen et al. [18] introduced an approach where head positions are encoded by first adding positional information, followed by head-specific position encoding. Building on this, we refined the method by incorporating matrix multiplication to capture spatial position information before integrating it into the attention heads’ positional encoding. Figure 4c presents our approach, which has been empirically validated to better capture spatial relationships, mitigate rank constraints on the attention matrix, and enhance the extraction of visual features.

The detailed steps are as follows:

First, absolute positional encoding (APE) is applied to both Q and K:

(1) $${pos}_{q}=APE\left(Q\right),{pos}_{k}=APE\left(K\right).$$

Next, spatial relationships within the attention head are computed by calculating the similarity between feature pixels:

(2) $${spatial}_{scores}={pos}_{q}.{pos}_{k^{T}}.$$

Finally, the spatial relationships are incorporated into the pixel similarity matrix. Equation (3) represents the calculation of the similarity matrix, and Equation (4) shows the fusion process:

(3) $$scores=\frac{Q\cdot K^{T}}{\sqrt{d_{k}}},$$

(4) $$scores=scores+{spatial}_{scores}$$

where $d_{k}$ denotes the scaling factor (the dimensionality of the key vector) and $K^{T}$ denotes the transpose of K.

#### 2.3.3. Learnable Sparse Mechanism (LSM)

In computer vision, algorithms such as Vision Transformer [19] and Swin Transformer [20] have made significant progress by leveraging the self-attention mechanism. These algorithms compute a similarity matrix by analyzing the correlation between pixels. They then refine the image information using a weight matrix obtained through scaling and batch normalization (BN), ultimately generating the output for each attention head. The overall process is as follows.

First, the model applies a linear transformation to the input feature, X, mapping it to three different representations: Q, K, and V:

(5) $$Q=XW_{Q},\;K=XW_{K},\;V=XW_{V}.$$

where $W_{Q},\;W_{K}$, and $W_{V}$ are the weight matrices.

Next, the attention mechanism calculates the similarity between Q and K:

(6) $$W_{O}=softmax\left(\frac{QK^{T}}{\sqrt{d_{k}}}\right)$$

where $W_{O}$ is the weight matrix for the output:

(7) $$head=Attention\left(Q,K,V\right)=softmax\left(W_{O}\right)\cdot V.$$

Finally, the outputs of multiple attention heads are concatenated and passed through a linear transformation to generate the final output:

(8) $$MultiHead\left(Q,K,V\right)=Concat\left({head}_{1},\ldots ,{head}_{h}\right)W_{O}.$$

where h denotes the number of attention heads.

**Figure 5:**
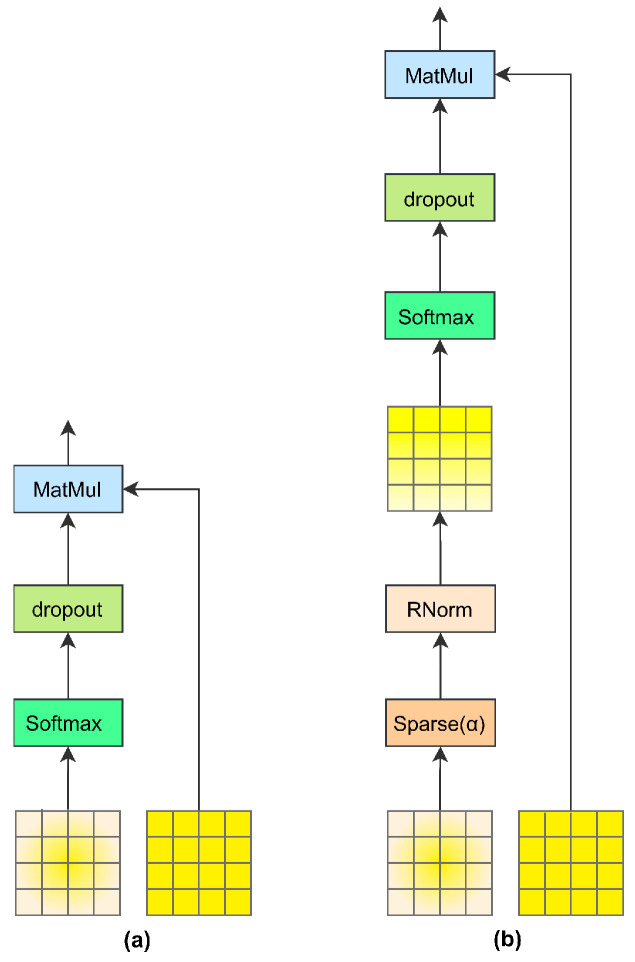
Comparison of sparse mechanisms. Note: (**a**) represents the flow chart of the standard Transformer processing and (**b**) represents the addition of the learnable sparse mechanism on this basis.

However, not all objects in poultry images exhibit strong correlations, which also applies to poultry representations. Computing pixel-wise similarity may introduce noise, compromising the accuracy of visual representations. In addition, traditional softmax normalization may fail to distinguish between objects in poultry images effectively. To overcome these challenges, inspired by Han et al. [21], we propose a learnable sparse mechanism that enhances the process in two key aspects. The overall workflow is illustrated in Figure 5, where Figure 5a represents the standard Transformer approach and Figure 5b highlights the improvements achieved by integrating the sparse mechanism.

First, after calculating the similarity in Equation (1) and incorporating head position data, small values in the weight matrix are discarded in Equation (4). At this step, we introduce a learnable hyperparameter, α, which determines the proportion of elements retained in the matrix, as defined by Equation (9):

(9) $$\alpha =\sigma \left(ration\right).$$

where σ is the sigmoid function, mapping the learnable parameter “ratio” to a value between 0 and 1.

Inspired by Li et al. [22], the initial value of α is set to 0.9 to retain the most important elements, and it is optimized during the training process alongside other model parameters. The retained elements form the set T, and for each element $S_{ij}$ in the matrix, if its weight is large and belongs to set T, it is kept; otherwise, it is assigned a value of negative infinity:

(10) $$S_{ij}^{\prime }=\left\{\begin{array}{ll} S_{ij}, & \;\mathrm{if}\;S_{ij}\in T\\ -\infty , & \;\mathrm{otherwise}\; \end{array}.\right.$$

This mechanism effectively reduces noise introduced by the self-attention mechanism, producing a sparse weight matrix, $S_{ij}^{\prime }$, with differentiated values.

Furthermore, the softmax function prioritizes features with higher similarity by assigning them greater weights, effectively filtering out irrelevant object information and enhancing the differentiation of visual features. To further optimize this process, we introduce the RNorm method, which integrates a learnable hyperparameter, γ (ranging from 0 to 1), into the conventional softmax function, allowing for adaptive control over feature weighting. To prevent γ from being too small, which would cause the model to focus solely on poultry while ignoring the surrounding environment, or too large, making it difficult to distinguish between important and less important features, the initial value of γ is set to 0.8 and is updated during training. The RNorm function is defined as follows:

(11) $$RNorm\left(x_{i}\right)=\frac{e^{\frac{x_{i}}{\gamma }}}{\sum_{j=1}^{N}e^{\frac{x_{j}}{\gamma }}}.$$

where γ is the scaling factor and $x_{i}$ and N represent the elements in the sparse similarity matrix and their total number, respectively.

Equation (11) calculates the exponential value for each element and divides it by the sum of all exponential values, yielding a probability distribution between 0 and 1. The hyperparameter, γ, adjusts the weight differences between features, ensuring that the sum of all probabilities equals 1. The resulting similarity matrix, $S_{ij}^{\prime \prime }$, is defined in Equation (12):

(12) $$S_{ij}^{\prime \prime }=RNorm\left(s_{ij}^{\prime }\right).$$

#### 2.3.4. Key Feature Enhancement (KFE)

**Figure 6:**
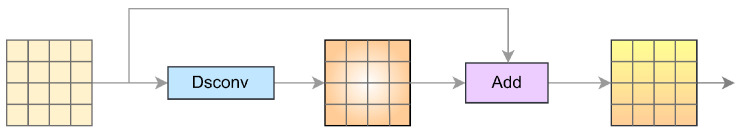
KFE process flow. Note: Dsconv stands for separable convolutional layers.

While Transformers outperform CNNs in capturing global contextual information, they can also introduce noise. Although the proposed learnable sparse mechanism (LSM) helps reduce this noise, it may still diminish the strength of local image features. To address this, we incorporate a depth-wise separable convolution layer, inspired by Pan et al. [23]. This addition preserves local features, and it also has local inductive bias, minimizing the complexity and maintaining computational efficiency. Figure 6 illustrates this process.

First, the global visual features (V) are processed through the depth-wise separable convolution layer, and the result is added back to the original V:

(13) $$V^{\prime }=Dsconv\left(V\right)+V.$$

where $V^{\prime }$ represents the enhanced matrix and Dsconv refers to the depth-wise separable convolution network.

Next, for each attention head, the output is calculated as follows:

(14) $${Output}_{\mathrm{CA}\_\mathrm{head}}=S_{ij}^{\prime \prime }\cdot V^{\prime }.$$

Finally, the outputs of all attention heads are concatenated to obtain the final MHCA output, as shown in Equation (8).

### 2.4. Multi-Level Attention Differentiator

Previous image captioning models have often focused heavily on visual feature extraction while overlooking the importance of the description process itself. In the PBC-Transformer, the classification task relies on visual features weighted by textual information, suggesting that the descriptive component is nearly as important as the visual features. Although deeper convolutional layers can extract global semantic features more effectively, these static vectors do not allow a model to dynamically focus on different regions to generate appropriate sentences [24]. This limitation is particularly evident in poultry behavior recognition, where images often contain numerous elements irrelevant to the poultry subjects or their behaviors. To address this issue, we propose a multi-level attention differentiator designed to refine attention by identifying multiple relevant regions within poultry images.

**Figure 7:**
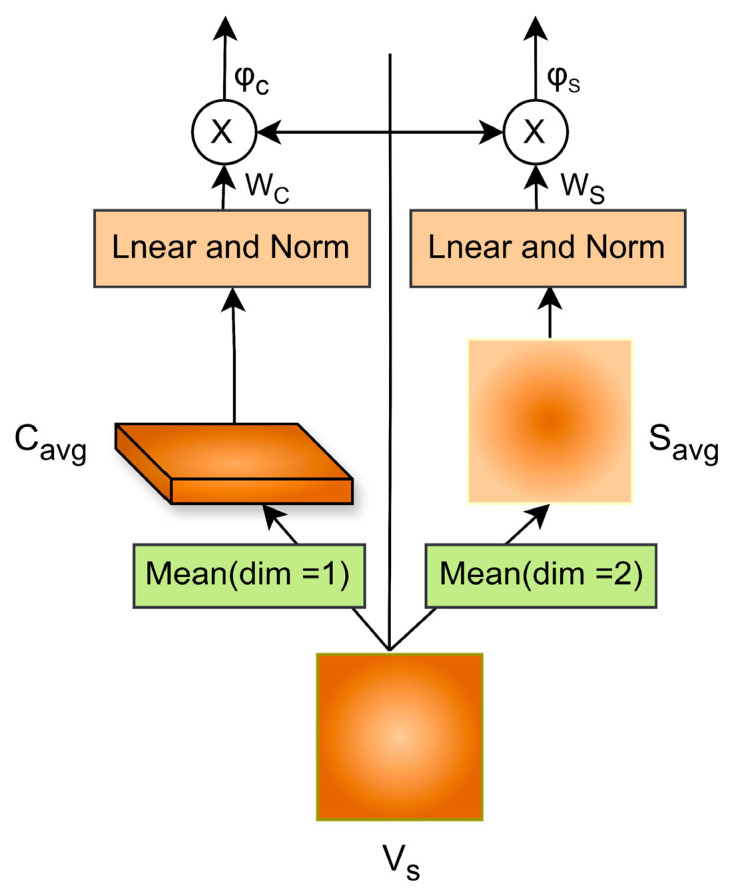
Multi-level attention differentiator. Note: The left direction is the process of processing features along the channel dimension, and the right direction is along the spatial dimension.

First, we extract a global semantic feature map ($V_{S}\in R^{B\times S\times C}$) from the feature encoder, where B, S, and C represent the batch size, spatial dimensions, and channels, respectively. Then, $V_{S}$ is further refined in both spatial and channel dimensions through the multi-level attention differentiator, as illustrated in Figure 7.

#### 2.4.1. Refinement of Spatial Features

Earlier image captioning models based on the CNN-LSTM architecture aimed to refine spatial attention [25], typically relying on the hidden state information of LSTM to focus on specific visual regions. In contrast, our spatial attention refinement method focuses on the spatial positions of poultry entities using only global features and their positional information. Specifically, the global semantic feature map, $V_{s}$, is aggregated along the spatial dimensions to generate a single feature map, $S_{avg}$, which represents the complete spatial information, as shown in Equation (15):

(15) $$S_{avg}=\frac{1}{C}\sum_{i=1}^{C}V_{s}\left(:,:,i\right).$$

where $S_{\mathrm{avg}\;}\in R^{B\times S\times 1}$.

Next,$S_{avg}$ is used to generate spatial attention weights through learned positional weights, which enhances the feature map by focusing on spatial regions relevant to poultry behavior, thereby providing spatial attention features ($\varphi_{S}$), as shown in Equation (16):

(16) $$\varphi_{S}=\sigma \;\left(W_{s}\cdot \;S_{\mathrm{avg}\;}\right)⊙V_{s}.$$

where $W_{s}$ is the learned parameter matrix for generating spatial attention weights and ⊙ denotes element-wise multiplication.

#### 2.4.2. Refinement of Channel Features

In global feature maps, each channel feature corresponds to the detector responses of the filters, which helps identify semantic information. Refining channel information allows the model to utilize channel attention features to filter parts of the poultry image relevant for behavior judgment. Specifically, $V_{s}$ is aggregated to extract channel descriptors ($C_{\mathrm{avg}}$), as expressed in Equation (17):

(17) $$C_{\mathrm{avg}\;}=\frac{1}{S}\sum_{k=1}^{S}V_{s}\left(:,K,:\right).$$

where $C_{\mathrm{avg}\;}\in R^{B\times 1\times C}$.

Subsequently, channel attention weights are obtained through learned weight parameters to capture scale diversity, which refines $V_{s}$ by focusing on entire spatial regions, resulting in channel attention features ($\varphi_{C}$), as shown in Equation (18):

(18) $$\varphi_{C}=\sigma \;\left(W_{c}\cdot C_{\mathrm{avg}}\right)⊙V_{s}.$$

where $W_{c}$ represents the learned parameter matrix for generating channel attention weights.

### 2.5. Memory Description Decoder

The purpose of the memory description decoder is to generate descriptive sentences corresponding to the visual features. It predicts the next word in the sentence based on the visual vectors. Formally, the input sentence is represented as $S_{i}^{\mathrm{in}}=\{[start],\ldots S_{1},\ldots S_{t}\ldots ,S.\left[end\right]\}$, where $S_{i}^{\mathrm{in}}\in R^{(T+1)\times L}$, T is the sentence length, and L denotes the vocabulary size. The special tokens [start] and $\left[end\right]$ are zero vectors. The output of the decoder is similarly represented as $S_{i}^{\mathrm{out}}=\{[start],\ldots S_{1},\ldots S_{t}\ldots ,S.\left[end\right]\}$.

**Figure 8:**
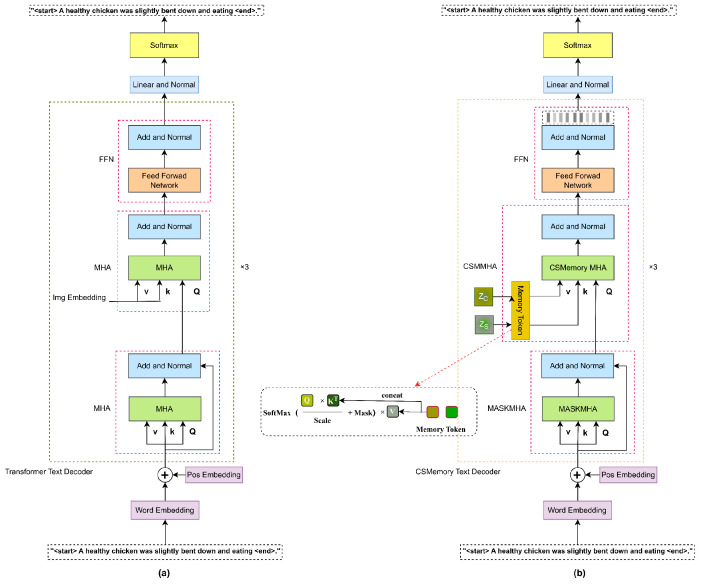
Description decoder comparison diagram. Note: (**a**) is the architectural diagram of the standard Transformer as a description of the decoder and (**b**) is the improvement we proposed on this basis by adding memory units in different dimensions.

The memory description decoder’s architecture mirrors the encoder’s, with three stacked standard Transformer layers. However, unlike the encoder, the decoder employs a masked multi-head self-attention mechanism (MASKMHA) to compute attention scores for each word, replacing the standard MHA with a memory multi-head self-attention layer (CSMMHA). Figure 8 compares the standard Transformer decoder (a) with the memory description decoder (b).

Overall Process:

First, we define learnable memory units in both the K and V dimensions for the refined spatial and channel attention, as described in Equations (19) and (20):

(19) $$K_{\mathrm{init}\;}=Parameter(randn\left(1,N_{\mathrm{memory}\;},d_{\mathrm{model}\;}\right)\times (\frac{1}{\sqrt{d_{\mathrm{model}\;}}})),$$

(20) $$V_{\mathrm{init}\;}=Parameter(randn\left(1,N_{\mathrm{memory}\;},d_{\mathrm{model}\;}\right)\times (\frac{1}{\sqrt{N_{\mathrm{memory}\;}}})).$$

where $N_{\mathrm{memory}\;}$ represents the number of memory units and $d_{\mathrm{model}}$ is the feature dimension.

Further, the memory units are connected to both the spatial and channel attention to serve as K and V for the memory multi-head self-attention, as shown in Equation (21):

(21) $$K_{\mathrm{final}\;}=\mathrm{cat}\left(K,K_{\mathrm{init}\;}\right),\;V_{\mathrm{final}\;}=\mathrm{cat}\left(V,V_{\mathrm{init}\;}\right).$$

Then, word embeddings, $S_{i}^{\mathrm{in}}$, are used as K, V, and Q inputs to the multi-head self-attention mechanism to calculate the attention scores, as expressed in Equation (22):

(22) $$Q_{\mathrm{final}\;}=AddNorm(MultiHead\left.\left(S_{i}^{\mathrm{in}},S_{i}^{\mathrm{in}},S_{i}^{\mathrm{in}}\right)\right).$$

The output, $Q_{\mathrm{final}}$, serves as the Q input for CSMMHA, implicitly exploring the relationships between the two modalities. Since the memory description decoder consists of three stacked Transformer layers, the output of each attention block becomes the input to the next block. Let $Ft_{i}$ represent the output of the final attention block, as defined in Equation (23):

(23) $$Ft_{i}=AddNorm\left(FFN\left(AddNorm(MultiHead\left.\left(K_{\mathrm{final}\;},V_{\mathrm{final}\;},Q_{\mathrm{final}\;}\right)\right)\right)\right).$$

Finally, the decoder uses a fully connected layer to transform $Ft_{i}$, creating a probability distribution for each token.

### 2.6. Classification Decoder

**Figure 9:**
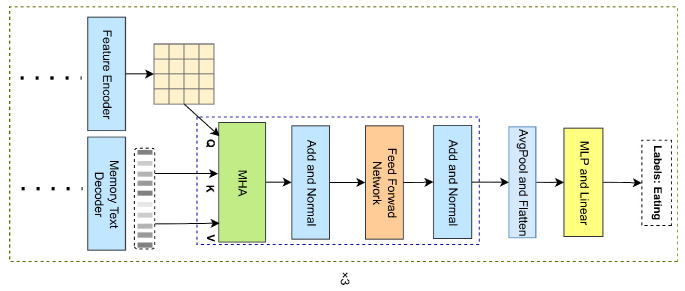
Classification decoder workflow. **Note:** Processing the visual features weighted by the text generated by the decoder. ‘x3’ indicates that there are three layers.

As shown in Figure 9, the classification decoder is designed to predict poultry behavior categories and shares a similar architecture with the encoder. In the description decoder, the output from each attention block is processed as a sequence of text-weighted visual feature vectors. These vectors are then used as K and V in the corresponding attention blocks of the classification decoder, while the global visual vector ($V_{s}$) serves as the Q. The overall process is as follows:

First, the sequence of text-weighted visual vectors generated by the description decoder is denoted as follows:

(24) $$H=\left\{h_{1},h_{2},\ldots ,h_{n}\right\}.$$

Next, the multi-head attention mechanism calculates the attention scores by computing the dot product between the Q and K, followed by normalization through the softmax function. The resulting values are then weighted and summed:

(25) $$A_{QKV}=\mathrm{CASMMHA}\left(Q_{cls},K_{cls},V_{cls}\right)=\mathrm{softmax}\left(\frac{Q_{cls}K_{cls}^{T}}{\sqrt{d_{k}}}\right)V_{cls}.$$

where $Q_{cls}=V_{s}$ and $V_{cls}=H$.

Finally, the predicted behavior category label, $y_{pred}$, is obtained through a multi-layer perceptron (MLP) and a linear layer:

(26) $$y_{pred}=softmax\left(\begin{array}{c} W_{1}\cdot Classifier\left(A_{QKV}\right)+MLP(A_{QKV})[:,0,:]) \end{array}\right).$$

where $W_{1}$ is the linear projection matrix that maps the attention mechanism output to the classification label space.

### 2.7. ICL-Loss (Image, Caption, and Label Loss Function)

In recent years, much focus has been centered on enhancing model architectures for image classification and caption generation, but the role of loss functions in model performance has been relatively overlooked [26,27,28]. Most models use cross-entropy loss to quantify the difference between predicted categories or generated captions and the ground truth. However, this approach primarily focuses on direct comparisons between predictions and target outputs, failing to fully capture the complex interrelationships among images, text, and labels. This limitation becomes more pronounced in multi-task learning scenarios. To tackle this, we propose incorporating contrastive learning into the loss function, proposing a triple contrastive learning loss function, ICL-Loss. This loss function leverages bimodal information from both vision and language and consists of three components: image mapping loss, text cross-entropy loss, and class cross-entropy loss.

Image mapping loss aligns image feature vectors into a new feature space via linear transformation, bringing image features semantically closer to corresponding text features in this space. Specifically, the image channel features are first aligned with the text features through a linear layer. Then, a subset of the mapped features is randomly selected to achieve image–text alignment:

(27) $$I_{\mathrm{selected}\;}=I_{\mathrm{mapped}\;}\left[S\right].$$

where $I_{\mathrm{mapped}\;}$ represents the linearly transformed image features and S is the randomly selected subset of image features.

Subsequently, the cosine similarity matrix, C, between the selected image features and text features is computed to measure their similarity:

(28) $$C\left(I_{\mathrm{selected}\;},T_{\mathrm{output}\;}\right)=\frac{I_{\mathrm{selected}\;}\cdot T_{\mathrm{output}\;}^{T}}{\left\|I_{\mathrm{selected}\;}\right\|||T_{\mathrm{output}\;}||}.$$

where $T_{\mathrm{output}\;}^{T}$ is the transposed text feature matrix.

The image mapping loss is then computed as follows:

(29) $$L_{\mathrm{image}\_\mathrm{to}\_\mathrm{text}\;}=\frac{1}{n}\sum_{i=1}^{n}\left[1-diag(C)]+max(C-margin,0)\right..$$

where diag(C) refers to the diagonal elements of the similarity matrix, representing the similarity of positive pairs. The margin is initially set to 0.2 to prevent convergence difficulties due to excessively large margins.

Text cross-entropy loss and class cross-entropy loss measure the match between the model-generated text sequences and categories with the target text sequences and true labels, respectively. These losses directly reflect the quality of the generated text and the accuracy of the category prediction:

(30) $$L_{\mathrm{text}\;}=\;\mathrm{CrossEntropy}(\mathrm{text}\_\mathrm{output},\;\mathrm{text}\_\mathrm{target}).$$

where text_output is the predicted text and text_target is the reference text.

(31) $$L_{\mathrm{class}\;}=\;\mathrm{CrossEntropy}(\mathrm{logits},\;\mathrm{labels}).$$

where logits are the predicted categories and labels are the true labels.

Before combining these three components, we introduce learnable weights to dynamically balance their contributions. Let $\lambda_{\mathrm{image}\;}$, $\lambda_{\mathrm{text}\;}$, and $\lambda_{\mathrm{class}\;}$ represent the weights for the image mapping loss, text cross-entropy loss, and class cross-entropy loss, respectively. Since image captions serve as auxiliary information to help the model and users better understand classification decisions, we initially set the weights to 0.4, 0.2, and 0.4, respectively, and adjust them during training through backpropagation. The total loss, L, is calculated as follows:

(32) $$L=\lambda_{\mathrm{image}\;}\cdot L_{\mathrm{image}\;}+\lambda_{\mathrm{text}\;}\cdot L_{\mathrm{text}\;}+\lambda_{\mathrm{class}\;}\cdot L_{\mathrm{class}}.$$

### 2.8. Dataset and Evaluation Metrics

#### 2.8.1. PBC-CapLabels (Poultry Behavior Classify—Caption Labels)

Poultry refers to domesticated birds, with chickens being the most widely raised species globally, followed by ducks and geese. Given the high visual and behavioral similarities among different poultry species, we selected four types of poultry, namely, chickens, ducks, geese, and pigeons, to construct a dataset of poultry behavior images with corresponding behavior descriptions named PBC-CapLabels.

**Figure 10:**
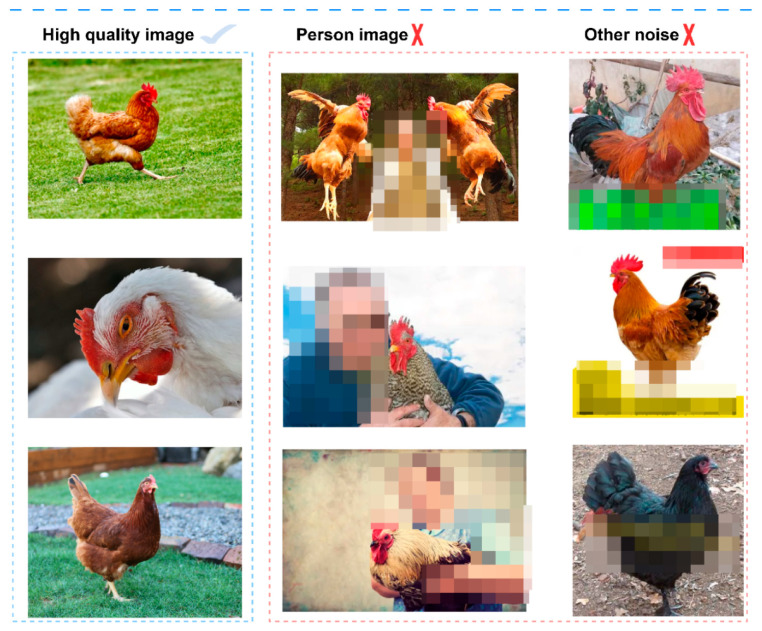
Examples of noisy data. Note: Noisy images containing some characters or text that contain ambiguities have been removed.

To enhance the robustness of the PBC-Transformer across diverse farming environments, data were collected through farm photography and web scraping. After obtaining permission from local farms and complying with relevant ethical guidelines, 10,000 candidate images were gathered. Low-quality or irrelevant images were removed, and the remaining images were standardized to JPEG format and resized to 224 × 224 pixels, discarding those below the resolution threshold. A pre-trained YOLOv5 model was applied to filter out images containing humans. With expert evaluation, 7179 high-quality images were selected for analysis. Examples of noisy images are shown in Figure 10.

For behavior categories, we classified avian behavior into nine subcategories: walking, running, standing, pecking, resting, grooming, flying, swimming, and abnormal behavior. These nine categories were used to evaluate the robustness of PBC-Transformer in handling imbalanced data. Furthermore, the dataset was further organized into three broader categories—movement, stationary, and resting—to assess the model’s performance under more balanced data conditions. The behavior classification references Fang et al.’s study on poultry behavior classification [29], as detailed in Table 1.

**Figure 11:**
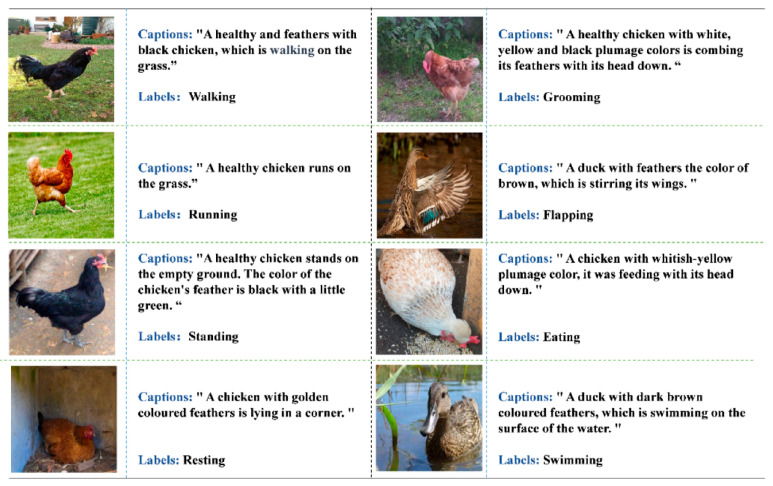
Examples from the PBC-Caplabels dataset. Note: It contains three components: behavioral images, behavioral categories, and behavioral descriptions.

For behavior descriptions, we adopted a format similar to the Microsoft COCO Captions dataset [30], with one description assigned per image. The dataset was randomly shuffled, and four agricultural experts provided descriptions for 5179 images. To reduce subjective bias, a pre-trained BLIP model [31] generated initial descriptions for the remaining 2000 images, which were then reviewed by experts. To prevent overfitting and improve the model’s robustness, natural noise (e.g., feather color and surrounding environment) was incorporated into the description dataset. An example of the dataset is shown in Figure 11.

#### 2.8.2. Flickr8K Dataset

**Figure 12:**
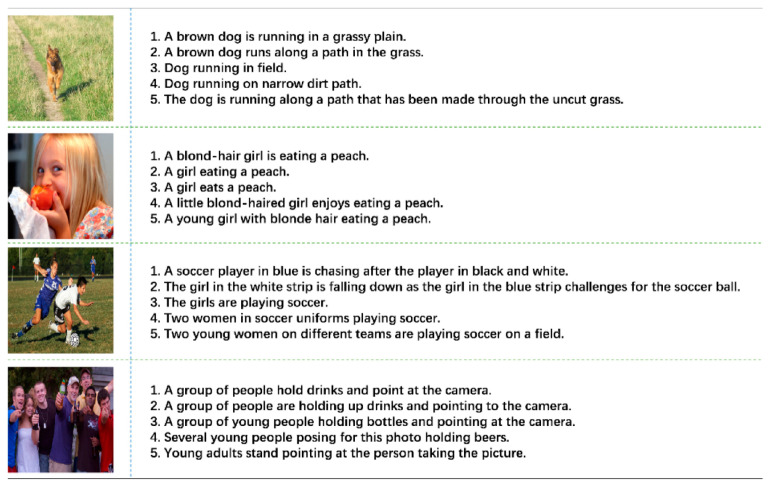
Examples from the Flickr8K dataset. Note: One image corresponds to four descriptions.

The Flickr8K dataset [32] is a key benchmark for image captioning, containing over 8000 images paired with five descriptive captions each. These images cover various scenarios, including personal activities, interactions, and environments. Examples from the Flickr8K dataset are shown in Figure 12.

#### 2.8.3. Evaluation Metrics

To evaluate caption generation performance, we use widely recognized metrics, including BLEU [33], ROUGE [34], Meteor [35], SPICE [36], and a composite metric, Sm. BLEU measures literal accuracy, ROUGE focuses on key information coverage, Meteor emphasizes contextual relevance, and SPICE prioritizes semantic accuracy and completeness. Given that poultry farmers are the end users, we prioritize semantic accuracy, key information coverage, and contextual relevance over literal accuracy, assigning different weights to these metrics in our composite metric, Sm, for evaluating poultry behavior description generation, calculated as follows:

(33) $$S_{m}=\frac{1}{10}BLEU_{4}+\frac{3}{10}\left({\;\mathrm{ROUGE}\;}_{L}+\mathrm{Spice}\;+\;\mathrm{Meteor}\;\right).$$

For the classification task, we use accuracy, recall, and F1 scores as evaluation metrics [37,38], defined as follows:

Accuracy: The percentage of correctly predicted samples out of the total:

(34) $$\mathrm{Accuracy}\;=\frac{TP+TN}{TP+TN+FP+FN}.$$

Recall: The probability of correctly identifying positive samples from actual positives:

(35) $$\mathrm{Recall}\;=\frac{TP}{TP+FN}.$$

F1 Score: The harmonic mean of precision and recall, balancing both:

(36) $$F1=\frac{2TP}{2TP+FN+FP}$$

where TP (true positive) and FP (false positive) refer to correctly and incorrectly predicted positive samples and FN (false negative) and TN (true negative) refer to incorrectly and correctly predicted negative samples, respectively.

## 3. Experiments

### 3.1. Overview

This study presents a method that utilizes image captioning to guide and interpret the decision-making process for poultry behavior classification. The PBC-CapLabels dataset is divided into two sets: a highly imbalanced nine-class set and a relatively balanced three-class set. The experimental design covers the following tasks:

- Comparative experiments between PBC-Transformer and traditional classification models.
- Comparative experiments between PBC-Transformer and conventional caption generation models.
- Ablation study of PBC-Transformer.
- Qualitative comparison of PBC-Transformer and other models on poultry behavior classification and description generation tasks.
- Generalization experiments of PBC-Transformer.

### 3.2. Experimental Setup

The experimental configuration and training parameters used in this study are summarized in Table 2.

For model construction, we utilized the pretrained EfficientNetB0 as the feature extractor and fine-tuned it during training. Following the approach of Liu et al. [39], we set the number of Transformer layers in both the encoder and decoder to three. During training, only the images, classes, and descriptions from the dataset were used, with no external data incorporated. The AdamW optimizer [40] was employed, and a dropout rate of 0.1 was applied to prevent overfitting. Greedy decoding was employed for sentence generation. All models, except the PBC-Transformer, were trained using the cross-entropy loss function.

### 3.3. Comparative Experiments

The comparative experiments assessed both the caption generation and behavior classification tasks. To ensure fairness and expedite model convergence, all models were pretrained on the ImageNet dataset and utilized identical parameter settings.

#### 3.3.1. Comparative Experiments on the Caption Generation Task

For the caption generation task, to demonstrate that the PBC-Transformer’s effectively addresses the first challenge (i.e., observation, attention, memory, description, and decision-making), we compared it with nine classic image captioning models, including both LSTM-based and Transformer-based methods. The compared models included the ShowTell model [14]: a traditional image captioning approach combining CNNs and LSTM; the Spitial model [40]: a model that incorporates attention mechanisms, allowing it to dynamically focus on image regions to generate more accurate captions; the FC model [41]: a model that uses CNNs to encode images and LSTM to predict the next word; the Att2in model [41]: a model that builds upon the FC model by dynamically assigning weights to image regions; the AdaAtt model [42]: a model that introduces a visual sentinel mechanism, enabling it to flexibly adjust its focus between image and textual features; the Att2all model [43]: a model that applies attention features to all gates of the LSTM, enhancing the coherence of the generated text; the AoANet model [44]: a model that utilizes an attention-over-attention mechanism, improving its ability to capture contextual information; the Grid model [45]: a model that employs a grid-based attention mechanism, enhancing caption accuracy by structuring image information into grids; and the M2 model [46]: a model that proposes a Meshed-Memory Transformer to enhance contextual information representation.

The results for the captioning task, presented in Table 3, were evaluated using metrics such as Bleu4, RougeL, Meteor, Spice, and Sm. Under identical parameter settings, the PBC-Transformer outperformed all other models in both the nine-class and three-class tasks. Notably, in the three-class task, it achieved a Bleu4 score of 0.493, surpassing the AdaAtt model by 0.057. In addition, the PBC-Transformer attained an Sm score of 0.588, which is 0.095 points higher than that of AdaAtt.

#### 3.3.2. Comparative Experiments on the Classification Task

In the classification task, to demonstrate that the proposed PBC-Transformer effectively addresses the second challenge (i.e., data imbalance, limited data, and integration of multimodal information), we compared PBC-Transformer with 13 classic classification models on both imbalanced (nine-class) and balanced (three-class) datasets. The models included VggNet19 [47], GoogleNet [48], InceptionV3 [49], AlexNet [50], DenseNet121/161 [51], ResNeXt50/101 [52], MobileNetV2 [20], EfficientNetB0/B2 [16], and SwinTransformerB/2B [20,53].

Table 4 presents the classification task results, showing that the PBC-Transformer achieved the highest performance, with an accuracy of 78.74% in the nine-class task and 80.70% in the three-class task. In the highly imbalanced nine-class scenario, it surpassed DenseNet by 7.66%, demonstrating its robustness in handling imbalanced data. In the three-class scenario, it outperformed GoogleNet by 5.81%, confirming its ability to leverage multimodal or multi-task learning to compensate for single-task weaknesses.

### 3.4. Ablation Study of PBC-Transformer

This study followed the poultry behavior classification process recommended by agricultural experts, systematically validating the rationale of integrating observation (CNN), attention (encoder), memory (memory unit), description (text decoder), and prediction (classification decoder). The results of the nine-class and three-class experiments are presented in Table 5 and Table 6.

Specifically, we employ EfficientNetB0 for the “Observation” stage. In Experiment Group A, a single convolutional layer partitioned the image into 20 × 20 or 40 × 40 grids (ensuring a fair comparison given EfficientNetB0’s 1280-dimensional output) and directly fed them into the classification decoder without incorporating any additional modules. Group B builds upon Group A by introducing the “Description” component, while Group C extends Group A with the “Observation” module. Group D further enhances Group C by incorporating “Description”, and Group E expands upon Group D with the addition of a “Memory” module. The results indicate that models incorporating the “Description” component outperform those without it across all evaluation metrics.

Performance further improves with the integration of specialized attention mechanisms (HSPC, LEM, and KFE) and the multi-stage attention differentiator, highlighting the model’s ability to capture subtle poultry behavior variations and address Challenge 3. Additionally, the inclusion of memory units (“Memory”) and text decoders (“Description”) significantly enhances both classification and captioning tasks, effectively addressing Challenges 1 and 2. The memory unit facilitates the integration of long-term behavioral patterns in a multi-task learning framework, refining caption generation and guiding classification decisions.

Finally, replacing traditional cross-entropy loss with ICL-Loss yields notable performance gains, particularly in Meteor scores, underscoring the model’s ability to capture contextual relationships. This suggests that cross-entropy loss may be suboptimal for multi-task learning, whereas ICL-Loss effectively optimizes task-specific loss relationships and weight allocation during training. This ablation study confirms that the PBC-Transformer successfully emulates the five core steps employed by agricultural experts in poultry behavior classification: Observation → Attention → Description → Memory → Prediction.

**Figure 13:**
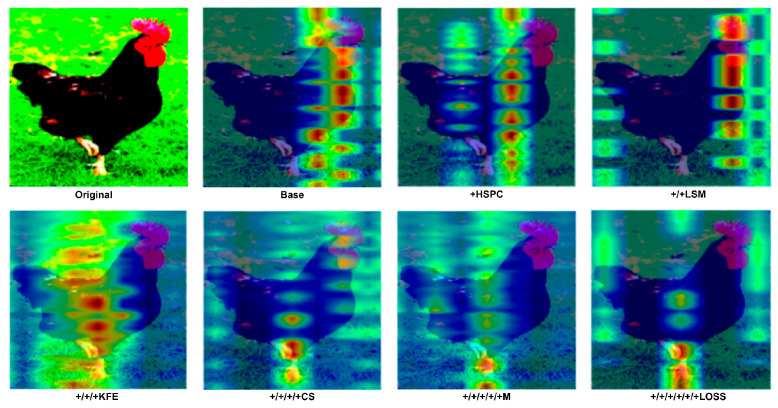
Attention visualization of the last layer in the classification decoder.

Additionally, we randomly selected a behavioral image from the validation set and visualized its attention map (using the weights from the nine-class dataset) to further explore the impact of each component on the model’s performance. Specifically, we visualized the attention output from the last layer of the classification decoder, as shown in Figure 13.

As shown in Figure 13, the classification decoder of the baseline model focuses most of its attention on the body of the chicken. After incorporating HSPC, irrelevant information or pixels unrelated to the chicken’s behavior are significantly filtered out, demonstrating the effectiveness of HSPC in optimizing the model’s self-attention mechanism. Furthermore, with the addition of LSM, aided by learnable parameters and the RNorm function, more irrelevant pixels are eliminated on top of the HSPC, enhancing the model’s ability to discard extraneous information. After adding KFE, the model’s ability to capture local features improves as it begins to focus on key body joints involved in chicken movement. In the final PBC-Transformer model, attention is predominantly focused on the primary joints associated with the behavior—specifically the feet—which serve as key indicators of walking behavior, as one foot is typically lifted during movement.. PBC-Transformer effectively eliminates most irrelevant elements compared to the baseline model. Although some minor irrelevant features remain, as mentioned earlier, this is intentional, introducing slight noise (such as background elements or feather color) during caption generation to prevent overfitting.

### 3.5. Qualitative Comparison

This section presents experimental tasks focused on qualitative comparisons for both the caption generation task and the behavior classification task.

#### 3.5.1. Qualitative Comparison on Caption Generation Task

To evaluate PBC-Transformer’s effectiveness in the captioning task, we performed a qualitative comparison against AOANet, Grid, and AdaAtt models. Since there was minimal variation between the nine-class and three-class models, only the weights from the nine-class model were used for inference.

**Figure 14:**
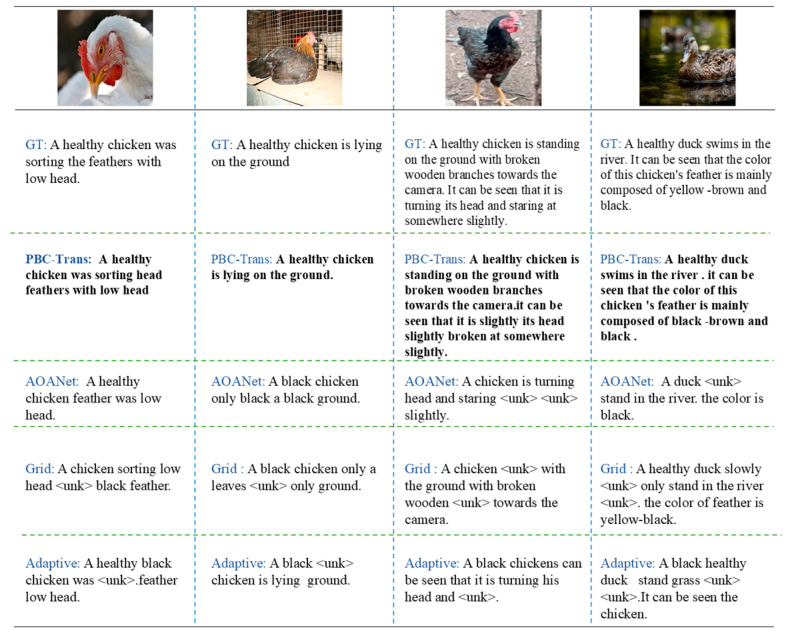
Captioning examples from PBC-Transformer and comparison models. Note: The words in black are the subtitle results predicted by PBC-Transformer, and the <unk> that appear are words that cannot be predicted by the model.

Figure 14 compares behavior descriptions generated by PBC-Transformer and the comparison models.

Taken together, existing models produced subpar captions, with almost all models generating the token <unk>, indicating difficulty handling low-frequency vocabulary. These models also lacked completeness in their descriptions. In contrast, PBC-Transformer excelled at predicting action-related words, producing readable and accurate captions even with noisy data, offering substantial guidance for behavior classification. Furthermore, PBC-Transformer consistently maintained caption completeness.

#### 3.5.2. Qualitative Comparison on Classification Task

**Figure 15:**
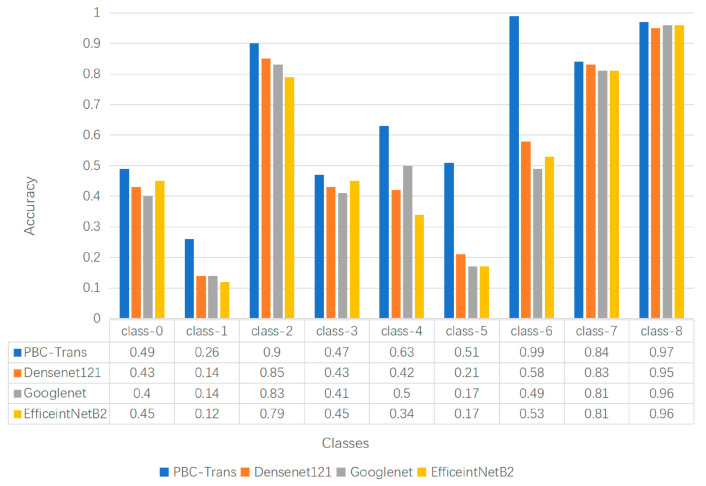
Qualitative comparison of classification results. Note: Predictive performance of different classification models in each category.

Figure 15 illustrates the performance comparison between the PBC-Transformer model and the comparison model on a 9-class classification task.. By leveraging multi-task learning and language-guided visual features, PBC-Transformer significantly outperformed traditional unimodal classification models.

It is noteworthy that in the five categories with limited sample sizes—Class 1 (215 samples), Class 3 (286 samples), Class 5 (145 samples), Class 6 (370 samples), and Class 7 (316 samples).PBC-Transformer significantly outperformed traditional classification models. In Class 6, PBC-Transformer achieved a perfect accuracy of 100%. This demonstrates that PBC-Transformer overcomes the limitation of conventional models, which typically require large sample sizes to achieve high performance. PBC-Transformer proves that excellent predictive performance can be attained even with a small number of samples.

**Figure 16:**
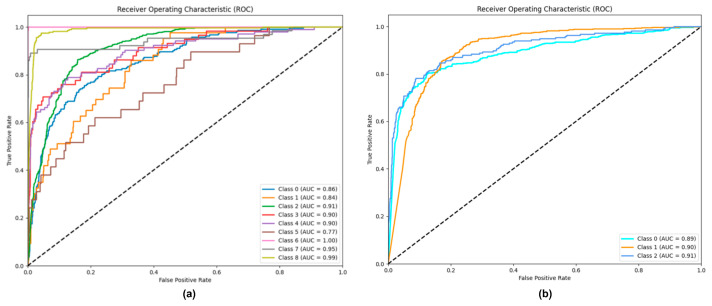
ROC curve of PBC-Transformer on the PBC-CapLabels dataset. Note: (**a**) illustrates performance in the nine-class classification task, while (**b**) represents the three-class classification results. The AUC values for each category are displayed in the bottom right corner of the figure.

We also present the ROC curves of the PBC-Transformer on the PBC-CapLabels dataset, as shown in Figure 16 (nine-class and three-class tasks).

In the nine-class scenario, the AUC values for most categories exceed 86%, demonstrating strong robustness and low false-positive rates. However, the AUC values for categories 1 and 5 are 84% and 77%, respectively, which can be attributed to the limited sample sizes (215 for category 1 and 145 for category 5), restricting the model’s learning capability. In the three-class scenario, all AUC values surpass 89%, reflecting the balanced distribution of samples and the resulting enhanced performance.

**Figure 17:**
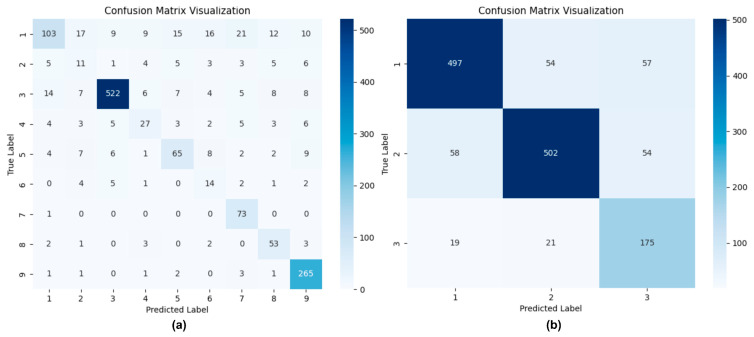
Confusion matrix visualization of PBC-Transformer’s inference results. Note: (**a**) represents the nine-class classification performance, while (**b**) corresponds to the three-class classification results.

Finally, we visualized the classification results of PBC-Transformer using confusion matrices for both the nine-class and three-class scenarios, as shown in Figure 17. Notably, PBC-Transformer demonstrates strong predictive capability even under highly imbalanced conditions in the nine-class dataset, with particularly pronounced accuracy in categories 5, 6, and 7.

### 3.6. Generalization Experiments

**Figure 18:**
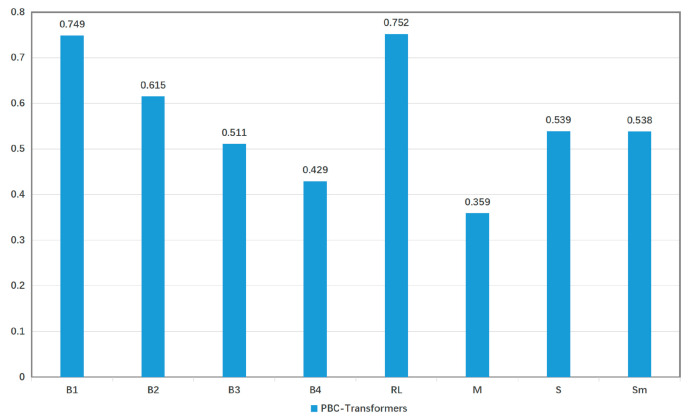
Performance of PBC-Transformer model on Flickr8k dataset.

This study explores the generalization capability of the PBC-Transformer model. Due to the absence of appropriate public datasets that encompass both captioning and classification tasks, we focus exclusively on evaluating the model’s performance in the captioning task as a proxy for its classification efficacy. Strong performance in captioning is expected to correlate with improved classification decision-making, driven by textually weighted visual features. We used the Flickr8k dataset, which has a similar volume of captions to PBC-CapLabels, for this generalization experiment. The results are presented in Figure 18.

The evaluation on the Flickr8k dataset confirms PBC-Transformer’s generalization ability and effectiveness across multiple metrics, demonstrating its practical applicability in diverse environments.

## 4. Results and Discussion

This study presents the PBC-Transformer model, designed to generate textual descriptions that explain poultry behavior classification results. We constructed the PBC-CapLabels dataset, which includes poultry behavior categories with corresponding descriptions, to evaluate the model. Throughout the research, we addressed three main challenges. The first challenge was to “mimic the process of agricultural experts in poultry Okinda behavior classification”. We integrated image captioning techniques into image classification tasks, using weighted visual features for classification. The PBC-Transformer not only generates classification results but also provides explanatory insights, following the expert process of Observation–Attention–Description–Recollection–Prediction. Based on this, we conclude that the PBC-Transformer effectively addresses this challenge. The second challenge was to “improve classification accuracy under conditions of limited data or imbalanced behavior categories”. Experimental results demonstrate that the PBC-Transformer achieved 78.74% accuracy in highly imbalanced data scenarios, outperforming the best-performing model by 7.66%, thus making significant progress in overcoming this challenge. The third challenge was to “filter out scenes unrelated to individual poultry or behaviors”. We addressed this by implementing a concentrated attention mechanism, a multi-level attention differentiator, a learnable sparse mechanism, and key feature enhancement, all of which were validated in ablation studies and successfully resolved this challenge.

## 5. Conclusions

We believe this work offers a novel perspective for the automated assessment of animal welfare. Beyond this specific application, the proposed technology is not limited to animal welfare research; we also believe it holds potential for other related agricultural domains, such as plant disease and pest identification.

Despite these achievements, the model still presents certain limitations. As a multi-task model, PBC-Transformer requires a multi-task dataset. Compared to traditional classification models, the inclusion of bimodal features leads to slower convergence and longer inference times, as well as higher demands on hardware resources. Furthermore, since part of the PBC-CapLabels dataset was collected online, it may not fully capture the diversity of real-world poultry farming environments, which could result in performance variability across different settings.

In future work, we aim to develop a more lightweight model that enhances performance while reducing training time, bringing it closer to traditional classification models, thus addressing the first limitation. We also plan to manually capture poultry behavior images from diverse farming conditions to expand the PBC-CapLabels dataset and address the second limitation.

## Figures and Tables

**Table 1:** PBC-CapLabels data distribution. Note: A dataset is divided into two different sets of categories: 9 categories and 3 categories.

9 Categories		
Categories	Description	Count
0: Walking	Moving slowly using feet	1057
1: Running	Moving quickly using feet	215
2: Standing	Standing still without movement	2902
3: Eating	Pecking behavior, usually with head lowered	286
4: Resting	Sleeping or resting on the ground	519
5: Grooming	Using beak to touch feathers	145
6: Abnormal Behavior	Non-routine, unusual behavior	370
7: Flapping	Flapping wings or feathers	316
8: Swimming	Moving through water	1368
**3 Categories**		
0: Movement	Includes walking, running, swimming, flapping, and some abnormal behaviors	3036
1: Stationary	Includes standing and some abnormal behaviors	3068
2: Resting	Includes pecking, resting, grooming, and some abnormal behaviors	1074

**Table 2:** Experimental configuration and parameters.

Experimental Configuration		Training Strategies	
Parameter	Value	Parameter	Value
GPU	RTX 4090	Pretrained	True
CPU	Intel 6230R	Epoch	200
OS	Windows	Batch Size	16
Pytorch	2.2.2	Optimizer	AdamW
Software	Python 3.8	Loss Function	Cross-Entropy
Cuda	12.2	Optimizer Alpha	0.9
Feature Extractor	EfficientNetB0	Optimizer Beta	0.999
Encoder Layers	3	Extractor	2 × 10^−4^
Decoder Layers	3	Encoder_lr	6 × 10^−5^
Num_heads	8	Decoder_lr	6 × 10^−5^
Embedding	1280	Learning Rate Annealing	Every 3 Cycles
Imgs_size	224 × 224	Annealing Rate	0.8×
Word Threshold	≥2	Early Stop	50
Word_size	1294	Droupt	0.1

**Table 3:** Results of the captioning comparison experiments. Note: Bolded results are for the best-performing model and underlined results are for the second-best-performing model. B4 is Bleu4, RL is RougeL, M is Meteor, S is Spcie.

Method	9-Class					3-Class				
	B4	RL	M	S	Sm	B4	RL	M	S	Sm
ShowTell	0.432	0.658	0.354	0.486	0.493	0.427	0.657	0.352	0.474	0.488
Spitial	0.415	0.644	0.345	0.466	0.478	0.410	0.641	0.343	0.463	0.475
FC	0.424	0.652	0.351	0.471	0.485	0.422	0.654	0.352	0.474	0.486
Att2in	0.420	0.644	0.347	0.460	0.477	0.404	0.628	0.338	0.445	0.464
AdaAtt	0.440	0.662	0.358	0.484	0.495	0.436	0.662	0.356	0.481	0.493
Att2all	0.405	0.628	0.338	0.449	0.465	0.408	0.631	0.340	0.453	0.468
AoANet	0.434	0.654	0.354	0.475	0.488	0.427	0.655	0.352	0.471	0.486
Grid	0.437	0.661	0.356	0.476	0.491	0.421	0.651	0.350	0.472	0.484
M2	0.435	0.660	0.354	0.482	0.492	0.429	0.658	0.354	0.475	0.489
**PBC-Trans**	**0.498**	**0.794**	**0.393**	**0.613**	**0.590**	**0.493**	**0.792**	**0.393**	**0.609**	**0.588**

**Table 4:** Results of the classification comparison experiments. Note: Bolded results are for the best-performing model and underlined results are for the second-best-performing model.

Method	9-Class			3-Class		
	Accuracy	F1	Recall	Accuracy	F1	Recall
VggNet19	68.23	66.99	68.23	73.79	73.56	73.79
GoogleNet	69.91	67.77	69.91	75.89	75.57	75.89
InceptionV3	62.55	58.92	62.55	67.55	66.84	67.55
AlexNet	65.04	62.02	65.04	67.71	67.14	67.71
DenseNet121	71.08	68.74	71.08	73.68	73.54	73.68
DenseNet161	69.83	66.75	69.83	73.04	73.19	73.04
ResNext50	68.86	66.57	68.86	73.71	73.03	73.71
ResNext101	66.78	63.00	66.78	72.88	72.15	72.88
MobieNetV2	67.00	65.38	67.00	73.54	73.27	73.54
EffcientNetB0	68.86	67.67	68.86	74.38	73.98	74.38
EfficientnetB2	69.83	68.28	69.83	75.50	75.28	75.50
SwinB	69.56	67.33	69.56	73.73	72.91	73.73
Swin2B	68.87	66.54	68.87	74.27	74.13	74.27
PBC-Trans	**78.74**	**83.94**	**78.74**	**81.70**	**81.14**	**81.70**

**Table 5:** Ablation results for the 9-class task. **Note:** ① represents HSPC; ② denotes the incorporation of LSM into ①; ③ includes KFE on top of ②. CSA refers to the multi-level attention differentiator. In the 9-class experiment, the model achieved optimal performance when the memory unit size was set to 30. Bolded results are for the best-performing model.

	Structure					Captions Indicators					Classify Indicators		
	CNN	Dec	CSA	N_m	Loss	B4	RL	M	S	Sm	Accu	F1	Recall
A	Class–Prediction (Grid petch 20 × 20 and 40 × 40)												
	×	×	×	×	×	——	——	——	——	——	43.61	31.61	43.61
	×	×	×	×	×	——	——	——	——	——	42.81	30.92	42.81
B	Class and Description–Prediction (Grid petch 20 × 20 and 40 × 40)												
	×	√	×	×	×	0.281	0.602	0.272	0.373	0.362	74.20	71.31	74.20
	×	√	×	×	×	0.334	0.659	0.301	0.435	0.413	72.89	70.28	72.89
C	Observation–Class–Prediction												
	√	×	×	×	×	——	——	——	——	——	68.86	67.67	68.86
D	Observation–Attention–Description–Prediction												
	√	√	×	×	×	0.407	0.742	0.348	0.530	0.526	76.23	74.02	76.23
	√	①	×	×	×	0.411	0.743	0.352	0.542	0.532	76.70	74.78	76.70
	√	②	×	×	×	0.421	0.752	0.354	0.545	0.537	76.71	75.21	76.71
	√	③	×	×	×	0.432	0.759	0.358	0.557	0.545	76.72	74.94	76.72
	√	③	√	×	×	0.442	0.767	0.366	0.564	0.553	76.78	75.16	76.78
**E**	Observation–Attention–Description–Recollection–Prediction												
	√	③	√	10	×	0.453	0.776	0.373	0.577	0.563	76.79	75.77	76.79
	√	③	√	20	×	0.460	0.776	0.379	0.582	0.567	76.80	74.95	76.80
	√	③	√	**30**	×	0.479	0.789	0.385	0.600	0.580	78.14	75.44	78.14
	√	③	√	40	×	0.472	0.787	0.382	0.596	0.576	77.06	75.04	77.06
	√	③	√	50	×	0.466	0.784	0.381	0.592	0.573	76.98	74.91	76.98
	√	③	√	30	√	**0.498**	**0.794**	**0.393**	**0.613**	**0.590**	**78.74**	**83.94**	**78.74**

**Table 6:** Ablation results for the 3-class task. **Note:** In the 3-category experiment, the model performed best when the memory unit was 40. Bolded results are for the best-performing model.

	Structure					Caption Indicators					Classify Indicators		
	CNN	Dec	CSA	N_m	Loss	B4	RL	M	S	Sm	Acc	F1	Recall
A	Class–Prediction (Grid petch 20 × 20 and 40 × 40)												
	×	×	×	×	×	——	——	——	——	——	52.31	47.71	52.32
	×	×	×	×	×	——	——	——	——	——	49.93	46.02	49.93
B	Class and Description–Prediction (Grid petch 20 × 20 and 40 × 40)												
	×	√	×	×	×	0.289	0.619	0.280	0.383	0.372	75.07	73.98	75.07
	×	√	×	×	×	0.355	0.682	0.311	0.457	0.432	74.38	74.42	74.38
C	Observation–Class–Prediction												
	√	×	×	×	×	——	——	——	——	——	74.25	73.88	74.25
D	Observation–Attention–Description–Prediction												
	√	√	×	×	×	0.408	0.736	0.344	0.528	0.523	79.15	79.15	79.15
	√	①	×	×	×	0.416	0.745	0.350	0.534	0.530	79.20	78.94	79.20
	√	②	×	×	×	0.424	0.747	0.355	0.544	0.536	79.62	79.50	79.62
	√	③	×	×	×	0.430	0.750	0.357	0.551	0.540	79.64	79.28	79.63
	√	③	√	×	×	0.440	0.759	0.362	0.556	0.547	79.70	79.46	79.70
E	Observation–Attention–Description–Recollection–Prediction												
	√	③	√	10	×	0.451	0.766	0.369	0.570	0.556	79.89	79.81	79.89
	√	③	√	20	×	0.460	0.773	0.375	0.574	0.562	80.03	79.86	80.03
	√	③	√	30	×	0.463	0.776	0.377	0.576	0.565	79.75	79.18	79.75
	√	③	√	**40**	×	0.477	0.785	0.385	0.593	0.577	80.24	80.14	80.24
	√	③	√	50	×	0.467	0.778	0.379	0.583	0.568	80.23	79.93	80.23
	√	③	√	30	√	**0.493**	**0.792**	**0.393**	**0.609**	**0.588**	**81.70**	**81.14**	**81.70**

## Data Availability

The data code is available online at: https://github.com/reinchow/PBC-Transformer (Accessed 24 May 2025). Because the image datasets occupy a large amount of storage, the datasets used and/or analyzed during the current study are available from the corresponding author upon reasonable request.
